# Effect of Mild Hypothermia after Craniotomy on the Function of Related Organs in Patients with Traumatic Brain Injury

**DOI:** 10.1155/2021/4105406

**Published:** 2021-10-08

**Authors:** Shu Cai, Zheng Lu

**Affiliations:** Department of Neurosurgery, Affiliated Haian Hospital of Nantong University, Haian 226600, Nantong, Jiangsu, China

## Abstract

**Objective:**

To investigate the effect of mild hypothermia after craniotomy on the function of related organs in patients with traumatic brain injury.

**Method:**

A total of 240 patients with craniocerebral injury from January 2017 to December 2020 were retrospectively analyzed. Patients were randomly divided into a control group and an experimental group, with 120 cases in each group. The control group was treated with craniotomy decompression, and the experimental group was treated with early mild hypothermia based on craniotomy decompression. Patients' venous blood was collected before operation (*T*_0_), at the end of operation (*T*_1_), 24 h after operation (*T*_2_), and 2 weeks after operation (*T*_3_) to detect the serum levels of the beta-subunit of S100 protein (S100-*β*); soluble growth stimulation expressed gene 2 (sST2), neutrophil gelatinase-associated lipocalin (NGAL), and interleukin 6 (IL-6). The prognostic effect was evaluated after 2 weeks of treatment.

**Results:**

After mild hypothermia treatment after craniotomy and decompression, the patients' serum S100-*β*, sST2, NGAL, and IL-6 levels at different time points were significantly lower than the control group, and the total effective rate was higher than that of the control group.

**Conclusion:**

The treatment of mild hypothermia after craniotomy can reduce the related organs function damage indicators and inflammatory stress response, thus improving clinical efficacy and prognosis.

## 1. Introduction

In recent years, with the continuous development of construction and transportation, the incidence rate of craniocerebral injury caused by external force directly or indirectly has increased. It is one of the common acute diseases in the Department of Neurosurgery [1–4]. The patients presented with headaches, dizziness, nausea, disturbance of consciousness, limb paralysis, etc. In severe cases, intracranial hematoma and brain hernia will occur and endanger life [5, 6]. Studies have shown that patients with craniocerebral injury have the characteristics of high mortality and high disability rate, and effective treatment can inhibit the progress of the disease and reduce mortality and disability rate [7, 8]. At present, craniocerebral injury mainly adopts conservative treatment and surgical treatment, but the clinical effect is unknown due to the lack of a unified treatment scheme [9, 10]. This study investigates the impact of mild hypothermia treatment after craniotomy decompression on patients with craniocerebral injury and its influence on related organs function indicators. The results are reported as follows.

## 2. Materials and Methods

### 2.1. General Information

Data were collected from 240 patients with craniocerebral injury admitted to the inpatient department of Affiliated Haian Hospital of Nantong University from January 2017 to December 2020 by retrospective analysis. Inclusion criteria: all patients were confirmed by CT or MRI examination, which met the diagnostic criteria of craniocerebral injury; the indications for craniotomy and decompression; Glasgow Coma Scale (GCS) below 8. Exclusion criteria: combined with other severe organ and body injury; coagulation dysfunction; contraindications for operation; a history of mental illness. The study was approved by the Ethics Committee of Affiliated Haian Hospital of Nantong University, and patients or their families signed informed consent.

### 2.2. Methods

240 patients were randomly divided into the control group and experimental group, 120 cases in each group. In the control group, only craniotomy and decompression were performed. The most common operation methods were as follows: scalp incision was made in front of the upper ear of the zygomatic arch, extending from the ear to the upper back and then cutting forward along the midline to the frontal hairline after reaching the parietal tubercle. The skin flap was free and the skull was exposed. An electric drill was utilized to make a bone hole on the surface of the skull, followed by milling the skull with the milling cutter to form a bone window and dealing with the hemorrhage of the dural artery and skull base. The dura mater was opened radially and suspended. Then, the intracranial hematoma and contused brain tissue were explored and cleared. Electrocoagulation was used for hemostasis, intracranial pressure monitoring probe was inserted, and finally the dura mater was reconstructed, and the skull was closed. The experimental group was treated early with a mild hypothermia therapy instrument based on craniotomy decompression: medical ice cap and blanket machine (Zhuhai Hokai Medical Instruments Co., Ltd., model and specification: HGT-200IV). After craniotomy decompression, the patients were treated with ice caps and ice blankets for continuous cooling, combined with lytic cocktail and muscle relaxant, such as promethazine, chlorpromazine, and Propofol. Ice bags can be used in the head and neck, armpit, groin, and other places. In these ways, the body temperature was controlled to 33-34°C. When the intracranial pressure dropped to the normal level for 24 hours, rewarming was started by increasing 1°C every 4 hours until the temperature returned to 37°C. The treatment time for mild hypothermia was about 5–13 days.

### 2.3. Observation Index

4 ml of the patients' venous blood was collected before operation (*T*_0_), at the end of operation (*T*_1_), 24 h after operation (*T*_2_), and 2 weeks after operation (*T*_3_). The samples were centrifuged at 3000 r/min for 10 min before the examination. The S100-*β*, sST2, NGAL, and IL-6 were detected. The test kit was purchased from Shanghai Abbott Biotechnology Engineering Co., Ltd. (Shanghai, China).

### 2.4. Evaluation of Prognosis

According to the Glasgow Outcome Scale (GOS) system, the prognosis of the two groups after 2 weeks of treatment was compared. Good: recovery of learning ability and working ability may be accompanied by mild dysfunction. Disability: mild disability but can live independently and work under protection. Severe disability: consciousness still exists, but language, cognition, and body function are seriously impaired. Plant survival: unresponsive, long-term coma, with a state of decerebrate or decortical rigidity. Death: cardiac arrest and respiratory arrest. Good recovery, mild disability, and severe disability are recognized as effective treatments, while plant survival and death are recognized as ineffective treatments.

### 2.5. Statistical Methods

SPSS 22.0 software was used for data processing. Measurement data were expressed as *x* ± *S*. *T*-test was used for statistical analysis. The counting data were expressed as (*n* (%)) and were analyzed by *χ*^2^ test. *p* < 0.05 was statistically significant.

## 3. Results

### 3.1. Comparison of General Information between the Two Groups

There were 57 males and 63 females in the control group, aged 20 to 68 years old, with an average of (42.08 ± 13.14). The causes of injury were traffic accidents in 57 cases, accidental fall injury in 20 patients, assault injury in 21 patients, and other 22 injury cases. There were 61 males and 59 females in the experimental group, aged from 21 to 65, with an average of (41.49 ± 14.31) years old. The causes of injury included 55 cases of traffic accidents, 15 cases of accidental falls, 25 cases of assaults, and 25 cases of other injuries. There was no significant difference in gender, age, and cause of injury between the two groups (*p* > 0.05). See Table 1.

### 3.2. Comparison of S100-*β*, sST2, and NGAL Levels between Groups of Patients at Different Time Points

Before treatment, there was no significant difference between S100-*β*, sST2, and NGAL serum levels (*p* > 0.05). After treatment, the serum levels of S100-*β*, sST2, and NGAL in the experimental group at different time points were significantly lower than those in the control group (*p* < 0.0001). See Tables 2–4.

### 3.3. Comparison of IL-6 Levels at Different Time Points between the Two Groups

Before treatment, there was no significant difference in serum IL-6 levels between the two groups (*p* > 0.05). After treatment, the serum IL-6 level of the experimental group at different time points was significantly lower than that of the control group (*p* < 0.0001). See Table 5.

### 3.4. Comparison of Prognosis between the Two Groups

After treatment, the total effective rate of the experimental group was 70.0% (84/120) which was higher than 54.2% (65/120) of the control group. The difference was statistically significant (*p* < 0.05). See Table 6.

## 4. Discussion

Brain injury is mainly caused by an external force, which is severe and rapid. If not treated in time, permanent dysfunction can occur in patients and even results in death for severe cases [11, 12]. The results showed that craniotomy could change the volume of the fixed intracranial space artificially. The operation can provide the most significant buffer space for intracranial hypertension caused by brain injury, which reduces intracranial pressure in time, maintains normal cerebral perfusion pressure, and improves brain oxygenation and metabolism [13, 14]. Mild hypothermia is considered as one of the most reliable physical therapy methods for patients with brain injury. It can reduce the brain metabolism and the toxic effects of harmful substances on neurons, such as excitatory amino acids and oxygen-free radicals. What's more, it can change the expression of apoptosis-related genes and finally inhibit the process of neuron apoptosis/necrosis [15, 16]. In this study, mild hypothermia after craniotomy was used to treat brain injury to clarify the intervention value on the function of related organs.

Patients with brain injury are prone to stress organ function injury after the operation, which is not conducive to recovering nerve function and reducing body trauma. The damage of brain, heart, and kidney greatly influences the patients. Patients with metabolic dysfunction and uncontrolled inflammatory response may lead to systemic inflammatory response syndrome (SIRS); eventually, it may result in multiple organ dysfunction syndrome (MODS). It is of great significance to reduce the damage of the above organs by positive treatment measures in the recovery of nerve function, maintenance of normal metabolism, stability of the internal environment, and control of the inflammatory response. Compared with the traditional biochemical indicators, such as S100-*β*, sST2, and NGAL have the characteristics of specificity, sensitivity and anti-interference ability. These indicators also have early diagnostic value in the diagnosis of brain, heart, and kidney function damage. In addition, the IL-6 level can effectively evaluate the severity of inflammatory response. So, we tested the serum levels of S100-*β*, sST2, NGAL, and IL-6 which were used to assess the function of the brain, heart, and kidney and the degree of stress inflammatory response after craniotomy.

S100-*β* is mainly expressed by astrocytes, and its content of normal body is minimal [17]. After brain injury, S100-*β* seeps out of the cell fluid and enters the cerebrospinal fluid and then into the blood through the damaged blood-brain barrier, leading to the increased concentration of S100-*β* in the blood. The moderate expression of S100-*β* is beneficial to the neurotrophic repair of the damaged parts. In comparison, the high level expression of S100-*β* shows neurotoxicity and induces apoptosis of the brain cells. So, S100-*β* is a biochemical marker that mainly reflects brain injury with high specificity and sensitivity. The higher the concentration of S100-*β* is, the worse the prognosis. Its expression level in serum is closely related to the degree of central nervous system injury and prognosis [18]. sST2 is one of the family members of the IL-1 receptor family. Two types of ST2 proteins directly affect the function of the heart: soluble ST2 and transmembrane ST2L. Soluble ST2, as the lure receptor of IL-33, can be competitive with IL-33, thus blocking the binding of IL-33 and ST2L and then weakening the cardiovascular protection of the IL-33/ST2L signaling pathway. A great quantity of soluble ST2 is produced during myocardial damage, making the heart lack enough IL-33 protection, which causes the heart to bear sustained pressure, cell death, and tissue fibrosis, resulting in heart function damage and even heart failure. sST2 is a new marker for evaluating cardiac function. It is not affected by age, renal function, or BMI [19]. NGAL is a new member of the lipocalin family. It is considered as one of the most effective biological markers in diagnosing acute renal injury. The early acute renal injury causes NGAL to be expressed in large amount in the blood. Serum NGAL will rise rapidly. So, NGAL can be used in the early diagnosis of renal function injury and reflect the severity of the injury. The expression level of NGAL in serum has a high diagnostic value to the disease assessment and prognosis judgment [20]. IL-6 is a multifunctional and effective cytokine that can regulate immune response and acute phase reaction. It plays an essential role in the body's inflammatory response. In the course of acute inflammation caused by trauma, surgery, stress reaction, and infection, IL-6 will be produced rapidly, which leads to the increase of serum IL-6 level, which can be used as early warning indicator. The IL-6 concentration of patients can predict whether complications will occur, and monitoring IL-6 levels in severe patients can effectively evaluate the degree of SIRS. The level of IL-6 also reflects the severity and prognosis of the disease [21, 22].

The results showed that after two weeks of mild hypothermia treatment for brain injury after craniotomy, the serum levels of S100-*β*, sST2, NGAL, and IL-6 were decreased in the experimental group. The results indicated that the mild hypothermia treatment after craniotomy could effectively improve organ function-related indicators after the traumatic stress response. The therapy could also inhibit the inflammatory response, reduce the further damage of related organs, and avoid SIRS and MODS. In conclusion, the treatment of mild hypothermia after craniotomy can promote the recovery of patients and improve the prognosis of patients, which is worth popularizing and applying in clinical practice in the future.

## Figures and Tables

**Table 1:** Comparison of general information between the two groups.

Groups	Cases	Sex (male/female)	Age (years)	Cause of injury			
				Traffic accident	Accidental fall injury	Assault injury	Others
Control	120	57/63	20–68 (42.08 ± 13.14)	57	20	21	22
Experimental	120	61/59	21–65 (41.49 ± 14.31)	55	15	25	25
*t*/*χ*^−2^		0.2667	0.3337	1.289			
*p* value		0.6055	0.7389	0.7317			

**Table 2:** The horizontal comparison of S100-*β* between two groups at different time points (*x* ± *S*, *μ*g/L).

Groups	*T*_0_	*T*_1_	*T*_2_	*T*_3_
Control	0.26 ± 0.13	0.17 ± 0.03	0.17 ± 0.03	0.16 ± 0.03
Experimental	0.27 ± 0.12	0.15 ± 0.01	0.13 ± 0.05	0.10 ± 0.03
*t* value	0.6192	6.928	7.515	15.490
*p* value	0.5364	<0.0001	<0.0001	<0.0001

**Table 3:** The horizontal comparison of sST2 between two groups at different time points (*x* ± *S*, *μ*g/L).

Groups	*T*_0_	*T*_1_	*T*_2_	*T*_3_
Control	33.34 ± 5.91	30.94 ± 6.97	24.57 ± 7.52	23.63 ± 8.53
Experimental	32.70 ± 6.62	28.65 ± 3.55	20.21 ± 8.12	15.42 ± 6.84
*t* value	0.790	3.207	4.316	8.226
*p* value	0.4303	0.0015	<0.0001	<0.0001

**Table 4:** The horizontal comparison of NGAL between two groups at different time points (*x* ± *S*, *μ*g/L).

Groups	*T*_0_	*T*_1_	*T*_2_	*T*_3_
Control	123.59 ± 6.87	116.66 ± 5.39	100.89 ± 10.32	82.36 ± 7.55
Experimental	122.85 ± 4.98	100.37 ± 7.66	83.99 ± 8.37	64.65 ± 5.79
*t* value	0.9554	19.05	13.93	20.23
*p* value	0.3404	<0.0001	<0.0001	<0.0001

**Table 5:** The horizontal comparison of IL-6 between two groups at different time points (*x* ± *S*, ng/L).

Groups	*T*_0_	*T*_1_	*T*_2_	*T*_3_
Control	15.79 ± 2.22	17.22 ± 2.25	13.69 ± 2.56	10.22 ± 2.25
Experimental	15.65 ± 2.42	13.53 ± 2.89	11.44 ± 2.50	8.33 ± 2.83
*t* value	0.4670	11.040	6.888	5.727
*p* value	0.6409	<0.0001	<0.0001	<0.0001

**Table 6:** Comparison of prognosis between the two groups (*n* (%)).

Groups	Good	Disability	Severe disability	Plant survival	Death	Total effective rate
Control	16 (13.3)	20 (16.7)	29 (24.2)	28 (23.3)	27 (22.5)	65 (54.2)
Experimental	39 (32.5)	27 (22.5)	18 (15.0)	17 (14.2)	20 (16.7)	84 (70.0)
*χ* ^2^ value						6.390
*p* value						0.0115

## Data Availability

The data used to support the findings of this study are included within the article.
